# Heavy Metal-Driven Oral Dysbiosis: Salivary Toxicometallomics at the Host–Microbiome Interface Across Pathologies

**DOI:** 10.3390/life16060920

**Published:** 2026-05-29

**Authors:** Claudia Florina Bogdan-Andreescu, Emin Cadar, Lucia Bubulac, Irina Anca Eremia, Viorica Tudor, Cristina-Crenguţa Albu, Iuliana-Raluca Gheorghe, Arsenie Dan Spînu, Andreea Mariana Bănăţeanu, Dan Alexandru Slăvescu

**Affiliations:** 1 Department of Specialty Disciplines, “Titu Maiorescu” University, 031593 Bucharest, Romania; claudia.andreescu@prof.utm.ro (C.F.B.-A.); andreea.banateanu@prof.utm.ro (A.M.B.); 2 Faculty of Pharmacy, “Ovidius” University, 900470 Constanța, Romania; emin.cadar@365.univ-ovidius.ro; 3 Department of Internal Medicine, Family Medicine and Labor Medicine, Faculty of Medicine, “Carol Davila” University of Medicine and Pharmacy, 050474 Bucharest, Romania; lucia.bubulac@umfcd.ro (L.B.); irina.eremia@umfcd.ro (I.A.E.); 4 Integrated Outpatient Department, “Prof. Dr. Agrippa Ionescu” Clinical Emergency Hospital, 011356 Bucharest, Romania; 5 Department of Genetics, Faculty of Dentistry, “Carol Davila” University of Medicine and Pharmacy, 020021 Bucharest, Romania; 6 Department of Complementary Science, Faculty of Medicine, “Carol Davila” University of Medicine and Pharmacy, 020021 Bucharest, Romania; raluca.gheorghe@umfcd.ro; 7 Department of Nephrology, Urology, Immunology and Immunology of Transplant, Dermatology, and Allergology, Faculty of Medicine, “Carol Davila” University of Medicine and Pharmacy, 020021 Bucharest, Romania; arsenie.spinu@umfcd.ro; 8 Department of Dentistry, Faculty of Medicine and Pharmacy, University of Oradea, 410073 Oradea, Romania; slavescudan@uoradea.ro

**Keywords:** heavy metals, salivary metallomics, oral microbiome, dysbiosis, biofilm ecology, metal resistance genes, oxidative stress, inflammation, environmental exposure, multi-omics biomarkers

## Abstract

Microbiome dysbiosis has become recognized as an important interface connecting environmental exposures to chronic inflammatory and degenerative diseases. Although prior research has largely considered heavy metals as biomarkers of exposure and toxicity, their function as ecological modulators of host-associated microbial communities remains underexplored. The oral cavity is a distinct exposome–microbiome interface where environmental, behavioral, and intraoral metal sources converge and interact with structured biofilms and mucosal immunity. This review adopts an ecological systems perspective, interpreting chronic low-dose exposure to metals such as cadmium, lead, mercury, nickel, chromium, arsenic, and aluminum as a sustained selective force on oral microbial networks. A resilience–threshold model is proposed in which cumulative metal pressure progressively diminishes microbial community stability, alters network topology, and drives transitions toward persistent dysbiosis. These modifications are further reinforced by oxidative–inflammatory feedback loops at the host–microbiome interface, facilitating a self-sustaining ecological imbalance. Sketching on insights from microbial ecology, environmental toxicology, and host response biology, this review presents a framework that links metallomic patterns to microbial restructuring, redox imbalance, immune activation, and regulatory adaptation. The analysis emphasizes ecological perturbations from stable dysbiotic states and identifies key methodological limitations that currently restrict causal inference. By conceptualizing heavy metals as active ecological drivers rather than passive exposure indicators, this work establishes a foundation for understanding microbiome-mediated disease susceptibility within an exposome-informed systems biology framework.

## 1. Introduction

Microbiome dysbiosis has been recognized as an important framework for understanding the connection between environmental exposures and chronic inflammatory, degenerative, and neoplastic diseases [1]. Rather than a simple compositional imbalance, dysbiosis involves modification of microbial community stability, characterized by reduced diversity, altered functional capacity, and impaired host–microbe signaling [2]. Accumulating evidence indicates that persistent low-dose environmental stressors can reshape microbial ecosystems, increasing disease susceptibility [3].

Representative published studies provide quantitative support for these associations. Davis et al. reported that low salivary concentrations of lead, cadmium, and arsenic were significantly associated with altered oral microbiome composition and increased dental caries prevalence in a population-based cohort, with metal-exposed individuals exhibiting measurable reductions in microbial diversity even at sub-threshold exposure levels [4]. Pei et al. demonstrated that residents living near a mining and smelting area displayed significant restructuring of oral microbial co-occurrence network topology relative to unexposed controls, with metal-exposed groups showing enrichment of stress-adapted and resistance-associated taxa [5]. Li et al. further reported that long-term heavy metal exposure in residents of mining and smelting regions was associated with a markedly higher abundance of oral antibiotic resistance genes, with co-localization of metal and antibiotic resistance determinants suggesting exposure-driven co-selection pressure [6]. These findings collectively indicate that chronic environmental metal burden exerts ecologically relevant effects on oral microbial diversity, community architecture, and resistance gene profiles. Furthermore, Zheng et al. demonstrated that co-exposure to heavy metals and smoking produced more pronounced disruption of oral microbial community structure than either exposure alone, suggesting that combined environmental burdens may exert synergistic effects on oral microbiome integrity [7].

Heavy metals are a distinct class of environmental stressors. Unlike transient chemical exposures, metals are non-biodegradable and bioaccumulative, exerting sustained biological pressure on host-associated microbiota [8]. Elements such as cadmium, lead, mercury, nickel, chromium, arsenic, and aluminum are prevalent in environmental, occupational, dietary, and intraoral dental appliances [9]. Although their systemic toxicological effects are well established, their function as modulators of microbial community structure and host–microbe interactions is only beginning to be clarified [10].

The epidemiological relevance of these interactions is substantial. According to the Global Burden of Disease Study 2019, approximately 1.1 billion people were affected by severe periodontitis worldwide—a figure nearly double that recorded in 1990—with periodontitis ranking as the 11th most prevalent disease globally and associated with an estimated annual productivity loss of USD 54 billion [11]. Oral cancer represents an additional dimension of this burden, accounting for approximately 355,000 cases annually and ranking as the 16th most prevalent malignant neoplasm globally, with heavy metals such as arsenic, cadmium, chromium, and lead recognized as carcinogenic agents that promote oral carcinogenesis through oxidative stress, DNA damage, and disruption of cellular repair mechanisms [12]. Regarding environmental metal exposure, lead alone was estimated to have caused 5.5 million adult deaths from cardiovascular disease and the loss of 765 million IQ points in children under five years globally in 2019, at an economic cost equivalent to 6.9% of global GDP [13]. Collectively, the high global prevalence of dysbiosis-associated oral diseases and the widespread environmental exposure to heavy metals underscore the need to investigate metals not solely as systemic toxicants but as ecological modulators of host-associated microbial communities.

Previous research has mainly investigated salivary heavy metals as biomarkers of exposure and disease processes, emphasizing their diagnostic and risk-stratification potential [14]. However, this approach does not fully address their impact on oral and systemic health. Besides serving as measurable indicators, metals may act as persistent selective forces that reshape microbial ecosystems and influence the host–oral microbiome. The oral cavity is a uniquely accessible and biologically dynamic interface between the exposome and the microbiome. It contains a highly diverse microbial ecosystem organized within structured biofilms and regulated by complex interactions among microorganisms, epithelial barriers, saliva, and immune mediators [15]. In this context, metals from systemic circulation, environmental deposition, lifestyle factors, or intraoral appliances are released into saliva and interact directly with biofilm architecture and mucosal surfaces [6]. Therefore, saliva should be considered not only a diagnostic fluid but also an ecological medium through which metal ions influence microbial competitiveness, redox balance, and inflammatory signaling [16].

Chronic low-dose exposure to heavy metals can progressively alter microbial community dynamics. Selective enrichment of metal-tolerant taxa, co-selection of antibiotic resistance determinants, disruption of quorum sensing (cell-to-cell microbial communication), and increased oxidative stress destabilize microbial networks and reduce ecological resilience [4]. As microbial stability declines, host-derived inflammatory responses further modify the local environment, creating feedback mechanisms that reinforce community imbalance and promote persistent dysbiosis [17].

Despite growing recognition of these interactions, most studies evaluate exposure, microbiome composition, and disease outcomes in isolation, limiting the development of integrated mechanistic models.

Within this context, salivary metallomics (comprehensive characterization of metal profiles within biological systems) attains broader biological significance. Beyond serving as an indicator of exposure, the salivary metallome may reflect the intensity of ecological pressure on the oral microbiome [10]. Convergent changes in microbial diversity, oxidative stress markers, inflammatory mediators, and epigenetic regulation may represent layered responses to sustained environmental stress [18].

Importantly, interpretation of salivary metallomic profiles requires consideration not only of total elemental concentration but also of chemical speciation, redox behavior, protein binding, and bioavailable fractions. These parameters determine the biologically active “ecological dose” encountered by microbial communities and host tissues and may partly explain variability across metallomic studies [19].

This review seeks to address these gaps by integrating environmental toxicology, microbial ecology, and host response biology within a systems-level framework. A resilience–threshold model is proposed, wherein cumulative metal exposure progressively reduces microbial network stability and facilitates the transition toward persistent dysbiosis. By conceptualizing the oral cavity as a sentinel ecological niche, this work reframes heavy metals from passive exposure indicators to potential ecological modulators of microbiome-associated pathology and underscores their relevance in shaping environmentally influenced disease susceptibility.

## 2. Metals as Ecological Stressors

Traditional toxicological models view heavy metals as dose-dependent cellular toxins that accumulate in the body. However, this approach misses their broader effects on host-associated microbial ecosystems. In the oral microbiome, heavy metals act as persistent ecological stressors rather than short-term toxins. These non-biodegradable metals accumulate in the environment over time [20]. At the microbial level, higher metal loads reduce community abundance, diversity, and function, signaling increasing ecological disruption [21].

Environmental stressors gradually reshape microbial ecosystems by reducing diversity and changing community structure. This supports the idea that microbiome responses are dynamic ecological processes, not just compositional shifts [22]. Microbiome resilience, the ability of microbial communities to resist or recover from disruption, is essential for maintaining stability under stress [23].

Prolonged heavy metal exposure creates selective pressure that favors organisms with resistance traits, such as efflux systems and detoxification pathways. This causes functional reorganization within the microbial community [9,24]. While microbes may initially adapt, resilience decreases over time, which can push the community toward a new, less stable state [25,26].

A key concept in this framework is the ‘ecological dose,’ which refers to the bioavailable, chemically reactive, and spatially accessible fraction of metals interacting with microbial communities over time, rather than a fixed toxicity threshold [27,28]. Biological impact depends on total concentration, persistence, speciation, and localization within biofilm microenvironments. Microorganisms mainly respond to labile metal fractions, with bioavailability affected by binding to salivary proteins and biofilm matrices [29,30]. In structured oral biofilms, extracellular polymeric substances create spatial gradients that influence metal distribution and microbial exposure, resulting in heterogeneous microenvironments [29,31]. Consequently, even low-level exposures can cause significant ecological effects when sustained across microbial generations, especially under chronic low-dose conditions [32]. This perspective shifts the focus from classical toxicity thresholds to cumulative ecological pressure and bioavailability-driven effects.

This transition fits within a resilience–threshold model. Below the threshold, disturbances are reversible and ecological stability is maintained. Once the threshold is crossed, microbial networks undergo structural and functional reorganization, resulting in persistent dysbiosis. Host-mediated processes such as oxidative stress and inflammatory signaling further reinforce this transition by altering the ecological niche.

Empirical support for these transitions is emerging. Davis et al. [4] demonstrated measurable reductions in oral microbial diversity even at sub-threshold salivary lead, cadmium, and arsenic concentrations, consistent with early-stage resilience erosion. Pei et al. [5] documented restructuring of oral microbial co-occurrence network topology in residents near mining areas, with metal-exposed groups showing enrichment of stress-adapted taxa—a pattern interpretable as threshold-proximal community reorganization. These findings illustrate how cumulative ecological pressure, rather than acute toxicity, drives the stepwise transitions described in the resilience–threshold model.

In this systems-level framework, heavy metals function as ecological modulators rather than direct pathogens, altering the conditions that shape microbial community function. Dysbiosis arises from sustained environmental pressure, driven by interactions among microbial adaptation, host responses, and physicochemical changes in the oral environment.

This conceptual framework guides the interpretation of the following sections, which integrate environmental exposure sources, bioavailability, and mechanistic pathways within an ecological model of metal-driven oral dysbiosis.

## 3. The Oral Cavity as an Exposome–Microbiome Interface

The oral cavity represents one of the most complex and continuously exposed ecological niches of the human body. Unlike internal organs that are shielded from direct environmental contact, the oral environment is simultaneously influenced by inhaled air, ingested substances, dental materials, behavioral habits, and systemic circulation [33]. This dual exposure situates the oral cavity at the intersection of the external exposome and internal physiological networks, making it a uniquely informative model for studying environmentally driven microbiome dysregulation [34].

### 3.1. Oral Biofilm as a Structured Ecological System

The oral microbiome is not a random assemblage of microorganisms but a highly organized, spatially structured ecosystem embedded within biofilms. These biofilms consist of multispecies consortia enmeshed in an extracellular polymeric substance (EPS) matrix, attached to enamel, dentin, mucosa, and restorative materials [33,35]. Within this structured architecture, microbial species engage in metabolic cooperation, competition, quorum sensing, and redox-based signaling [36]. Keystone taxa maintain ecological balance by regulating nutrient flux, pH homeostasis, and community stability [37].

Ecological equilibrium within oral biofilms depends on tightly regulated physicochemical parameters, including pH, oxygen gradients, salivary flow, nutrient availability, and host immune modulation [38]. Even subtle perturbations in these parameters can shift microbial community composition and functional output. Environmental toxicants, including heavy metals, therefore act not merely as chemical contaminants but as ecological modifiers capable of altering selective pressures within this structured microbial network [32].

### 3.2. Saliva as an Ecological Medium

Saliva is traditionally viewed as a diagnostic biofluid; however, from an ecological perspective, it functions as a dynamic medium that continuously bathes oral biofilms and epithelial surfaces [39]. It regulates hydration, buffering capacity, nutrient transport, antimicrobial peptide delivery, immunoglobulin distribution, and mechanical clearance of microorganisms. Its composition is shaped by salivary gland secretion, systemic diffusion, dietary components, and intraoral material dissolution [40].

Heavy metals enter saliva through multiple convergent pathways: systemic circulation via salivary gland secretion, direct dissolution from metallic dental materials, environmental deposition, and lifestyle-related exposures such as tobacco or aerosolized particles [14,41]. Once present in saliva, metal ions interact directly with the biofilm matrix, microbial cell walls, and salivary proteins. Their bioavailability is modulated by pH, redox state, protein binding capacity, and flow rate, all of which influence their ecological impact [42].

Thus, saliva should be understood not only as a carrier of exposure signals but as an active ecological compartment in which metals participate in chemical reactions, influence redox dynamics, and alter microbial survival and competitiveness.

### 3.3. Host–Microbe–Metal Interactions

The oral exposome–microbiome interface is defined by a dynamic triad involving host tissues, microbial communities, and environmental agents such as heavy metals [43]. Within this framework, metals exert effects at multiple levels: they impose selective pressure on microbial communities, favoring resistant taxa and destabilizing community structure; they amplify oxidative stress in host tissues, compromise epithelial barrier integrity, and modulate immune signaling pathways; and they alter the physicochemical properties of saliva and biofilms, thereby reshaping microbial metabolism and interspecies communication [44].

Importantly, these effects are interdependent and frequently self-reinforcing. Metal-induced oxidative stress within epithelial cells can modify nutrient availability and inflammatory mediators in saliva, which in turn reshapes microbial composition and biofilm function [45]. Conversely, dysbiotic microbial communities may shift local pH and redox balance, increasing metal solubility and retention and thereby intensifying ecological perturbation [46]. Through this bidirectional coupling, transient exposure signals can be converted into sustained ecological imbalance at the host–microbiome interface.

### 3.4. The Oral Cavity as a Sentinel Ecological Niche

Because of its accessibility, rapid cellular turnover, dense microbial colonization, and continuous environmental exposure, the oral cavity may function as a sentinel niche for detecting early ecological consequences of environmental metal burden [20]. Dysbiotic shifts within oral biofilms can emerge before overt clinical manifestations of disease and may reflect broader host–environment interactions [42].

Within this context, the concept of an “oral metallobiome” becomes particularly relevant: a microbial ecosystem shaped by chronic exposure to trace metals that influence microbial composition, functional pathways, and host signaling. Understanding this interface provides insight into how persistent environmental toxicants contribute to microbiome-driven pathologies and highlights the need to integrate environmental toxicology with microbial ecology in the study of chronic disease mechanisms.

## 4. Environmental and Intraoral Sources of Ecological Metal Pressure

Heavy metals enter the oral ecosystem through multiple, often overlapping pathways that collectively define the intensity and persistence of ecological pressure acting upon microbial communities [47]. From a microbiome-centered perspective, these sources do not merely increase measurable salivary concentrations; they introduce sustained physicochemical stress capable of reshaping biofilm structure, redox dynamics, and microbial network stability [48]. The magnitude, chronicity, and chemical speciation of exposure determine whether metals function as transient perturbations or long-term selective forces within oral biofilms [18].

### 4.1. Environmental and Occupational Exposure

Industrial emissions, traffic-related air pollution, mining activities, waste incineration, and contaminated water or soil contribute to widespread exposure to metals such as lead (Pb), cadmium (Cd), arsenic (As), mercury (Hg), chromium (Cr), and nickel (Ni) [49]. Following inhalation or ingestion, these elements enter systemic circulation and may be partially secreted into saliva via salivary glands, thereby reaching the oral biofilm environment [4].

Even when present at concentrations below classical systemic toxicity thresholds, persistent low-dose exposure may exert ecological pressure within oral communities. Metal ions can bind to extracellular polymeric substances, alter ionic gradients, and modulate local redox conditions, subtly reshaping microbial competitiveness [50]. Over time, sustained exposure may reduce alpha diversity, destabilize co-occurrence networks, and favor organisms harboring resistance mechanisms such as efflux pumps or metal-binding proteins [51]. Occupational settings intensify this process by increasing exposure frequency and cumulative burden, thereby accelerating microbial adaptation and enrichment of resistant phenotypes [52].

### 4.2. Lifestyle-Related Exposures

Lifestyle behaviors represent dynamic and repetitive sources of intraoral metal exposure. Tobacco smoke delivers bioavailable cadmium, lead, arsenic, and nickel directly to oral surfaces, creating cyclical deposition events that interact with smoking-induced reductions in salivary flow and altered pH [53]. Electronic cigarette aerosols may similarly introduce trace metals released from heating components, sustaining localized metal accumulation within saliva and biofilms [54].

Dietary intake provides an additional exposure pathway, particularly in regions with contaminated groundwater or food sources [55]. Acidic beverages may enhance intraoral metal solubility—especially from dental materials—thereby increasing ionic availability within plaque microenvironments [56]. In these contexts, ecological relevance arises not from isolated exposure episodes but from repeated micro-exposures that maintain selective pressure across microbial generations.

### 4.3. Dental Materials and Localized Metal Release

The oral cavity uniquely serves not only as an exposure interface but also as a direct source of metal release. Metallic restorations, orthodontic appliances, prosthetic alloys, and implant components may undergo corrosion, wear, or galvanic interactions under fluctuating pH and enzymatic conditions [57,58]. Metallic and polymeric restorative materials may influence local oral microenvironments through corrosion processes, ion release, surface physicochemical properties, and biofilm interactions. Consequently, there is increasing interest in the development and clinical application of modern polymer-based restorative materials as alternatives to conventional metallic systems [59,60,61,62]. Ion release from such materials introduces spatially localized and often prolonged metal pressure within adjacent biofilm niches [63].

Acidic microenvironments—such as those present in cariogenic plaque—enhance metal solubility and bioavailability, reinforcing localized selective stress [64]. Unlike systemic exposure, intraoral release establishes microhabitat-specific gradients within biofilms, fostering niche adaptation and enrichment of subpopulations capable of tolerating or metabolically adapting to metal stress [65]. In susceptible individuals, this localized ecological pressure may interact with immune responses and inflammatory signaling, contributing to dysbiotic restructuring [66].

### 4.4. Mixed and Cumulative Exposure Patterns

In real-world settings, exposure rarely originates from a single source. Environmental contamination, occupational contact, lifestyle behaviors, and dental materials frequently coexist, generating mixed-metal profiles within saliva [40,67]. These combinations may exert additive or synergistic ecological effects, intensifying selective pressure and promoting co-enrichment of multi-resistance determinants [68].

Importantly, chronic low-level mixtures may operate below classical toxicity thresholds while still destabilizing microbial network architecture [69]. Because microbial communities respond to integrated environmental stress rather than isolated agents, cumulative exposure patterns are particularly relevant for understanding transitions from ecological resilience to dysbiosis [70].

Collectively, these environmental and intraoral pathways define the cumulative metal stress landscape shaping oral microbial communities [5]. The persistence, mixture complexity, and bioavailability of exposure determine whether the oral ecosystem maintains adaptive stability or shifts toward a less resilient, dysbiosis-prone configuration [71].

The principal heavy metals implicated in oral ecological destabilization, together with their major exposure sources, microbiome-level effects, host responses, and dysbiosis-associated pathological implications, are summarized in Table 1.

## 5. Salivary Metal Bioavailability and Ecological Dose

The ecological impact of heavy metals within the oral cavity depends not solely on total concentration, but on bioavailability, chemical speciation, retention dynamics, and interaction with biofilm microenvironments [14]. From a microbiome-centered perspective, the concept of “ecological dose” is more relevant than systemic burden: it reflects the fraction of metal that is chemically active, spatially accessible to microorganisms, and persistent within the salivary–biofilm interface [18].

### 5.1. Entry into Saliva and Local Availability

Heavy metals reach saliva through two principal routes: systemic secretion via salivary glands and direct intraoral release from environmental deposition or dental materials [8,72]. Once present in saliva, metals may exist as free ionic species, weakly protein-bound forms, or components of larger complexes. The proportion of these fractions determines their interaction potential with microbial cells and biofilm matrices [73].

Free or loosely complexed ions are generally more biologically reactive. They can interact with bacterial cell membranes, bind to enzymatic cofactors, interfere with redox systems, and alter intracellular metal homeostasis. In contrast, strongly protein-bound metals may serve as slower-release reservoirs, prolonging low-level exposure within the oral ecosystem [29].

Salivary flow rate critically modulates ecological exposure. Reduced salivary flow, as observed in xerostomia or inflammatory conditions, concentrates dissolved ions and prolongs their contact time with biofilms. Conversely, high flow may dilute metal concentrations but can also facilitate continuous redistribution across oral surfaces [74,75].

Consequently, total salivary metal concentration alone may inadequately reflect the fraction that is microbiologically or immunologically active. Differences in bioavailability, ligand binding, local pH, salivary flow, and physicochemical microenvironments may substantially influence ecological impact, complicating direct comparison across studies [4,19].

### 5.2. Chemical Speciation and Redox State

The biological behavior of metals is strongly influenced by chemical speciation. For example, inorganic mercury (Hg^2+^) differs markedly from organic mercury species in membrane permeability and protein affinity [76]. Arsenic species (As^3+^, As^5+^, methylated forms) exhibit distinct redox properties and toxicity profiles [77]. Chromium, particularly in its hexavalent form (Cr(VI)), can penetrate cells more readily than its trivalent counterpart [78].

Within saliva, speciation is shaped by pH, oxidative status, enzymatic activity, and microbial metabolism. Acidic microenvironments—common in cariogenic plaque—enhance metal solubility and increase the fraction of bioavailable ions [79]. Redox-active metals may undergo valence changes within the biofilm, generating reactive oxygen species that further modify both microbial physiology and metal reactivity [80].

These transformations mean that salivary metals are not static chemical entities; rather, they participate dynamically in redox cycles that influence microbial survival and competitive fitness [4].

Collectively, these observations support the concept that biological interpretation of salivary metallomics should extend beyond absolute concentration values and incorporate speciation-dependent ecological activity.

Metal speciation may also influence microbial adaptive responses by altering selective pressure intensity, metal uptake, and resistance pathway activation [81]. Consequently, chemically distinct forms of the same metal may produce substantially different ecological effects despite similar total concentrations [82].

### 5.3. Interaction with the Biofilm Matrix

Oral biofilms provide a heterogeneous microenvironment composed of extracellular polymeric substances (EPS), bacterial cell surfaces, salivary proteins, and host-derived molecules [30]. The EPS matrix contains negatively charged components capable of binding cationic metal ions, effectively acting as a localized reservoir [83].

Metal accumulation within the matrix may produce concentration gradients across biofilm layers. Surface-exposed microorganisms may experience higher ionic stress, while deeper layers encounter delayed or buffered exposure. This spatial heterogeneity fosters micro-niches that promote selective adaptation rather than uniform toxicity [84].

In addition, metals can alter the physicochemical properties of the EPS matrix itself. Cross-linking with polysaccharides or proteins may modify biofilm rigidity, permeability, and diffusion dynamics. Such structural changes can influence nutrient transport, oxygen gradients, and intercellular signaling, thereby reshaping ecological balance [85].

### 5.4. Retention, Persistence, and Cumulative Ecological Exposure

Unlike transient chemical toxicants that are rapidly metabolized or eliminated after short-term exposure, heavy metals are characterized by environmental persistence and long-term bioaccumulation [86]. Even when salivary concentrations fluctuate in response to recent exposure, chronic low-level input may maintain a baseline ecological pressure [87].

Re-adsorption to enamel, mucosal surfaces, and plaque biofilms can prolong local retention. Swallowing does not necessarily terminate exposure, as continuous secretion and intraoral release sustain a dynamic equilibrium [88]. In this context, ecological dose reflects cumulative interaction over time rather than isolated peak values.

Repeated micro-exposures—through smoking, dietary intake, environmental air, or corrosion of dental materials—may therefore exert long-term selective pressure despite individually low concentrations [89]. This sustained low-dose exposure is particularly relevant to microbiome adaptation, as microbial communities respond to chronic stress by selecting for resistant phenotypes and reorganizing network structure [90].

### 5.5. Ecological Implications

Taken together, salivary bioavailability, chemical speciation, matrix binding, and retention dynamics determine the functional impact of metals on oral microbial communities. The concept of ecological dose emphasizes that dysbiosis may arise not from overt toxicity but from persistent, subclinical perturbations that gradually reshape microbial composition and host–microbe signaling [91].

Understanding these determinants is essential for interpreting how environmental metal burden translates into microbiome imbalance and, ultimately, disease susceptibility.

## 6. Mechanisms of Metal-Driven Oral Dysbiosis

Heavy metals contribute to oral pathology not primarily through acute cytotoxicity, but through sustained ecological modulation of microbial communities. At subclinical concentrations, metals act as selective forces that reshape microbial diversity, functional capacity, interspecies communication, and host–microbe signaling [5]. The transition from ecological perturbation to dysbiosis reflects a gradual reorganization of the biofilm ecosystem under chronic physicochemical stress [92].

### 6.1. Selective Pressure, Diversity Loss, and Network Destabilization

Microbial communities maintain stability through redundancy, metabolic cooperation, and complex interaction networks. Heavy metals impose selective pressure that disproportionately affects metal-sensitive taxa while permitting expansion of resistant or stress-adapted organisms [43].

Even low concentrations of cadmium, lead, nickel, chromium, or mercury can reduce alpha diversity within oral biofilms. Decline of keystone commensals may destabilize metabolic cross-feeding networks and disrupt network-level buffering mechanisms. As diversity decreases, microbial communities often shift toward configurations enriched in opportunistic, inflammation-associated taxa [93].

Network analyses from environmental microbiology suggest that metal exposure weakens co-occurrence interactions and reduces ecological resilience. In oral ecosystems, this may translate into reduced resistance to secondary stressors such as pH fluctuations, dietary sugars, or immune activation. The result is a transition from a stable, symbiotic biofilm toward a dysbiotic configuration characterized by functional imbalance rather than simple overgrowth of a single pathogen [94,95].

Importantly, dysbiosis in this context is not defined solely by compositional shifts, but by altered network topology and reduced capacity for self-regulation [70].

### 6.2. Metal Resistance Determinants and Co-Selection of Antibiotic Resistance

A critical mechanism underlying metal-driven dysbiosis is the enrichment of metal resistance genes (MRGs). Many bacteria harbor operons such as *czc* (cadmium–zinc–cobalt resistance), *cop* (copper resistance), *ars* (arsenic resistance), and *mer* (mercury resistance), which encode efflux pumps, sequestration proteins, and detoxification enzymes [96].

Under chronic exposure, taxa possessing these determinants gain a competitive advantage. Over time, the relative abundance of MRG-harboring organisms increases, reshaping the functional gene pool of the biofilm [97].

Notably, metal resistance genes are frequently co-located with antibiotic resistance genes on plasmids and transposons. This physical linkage allows environmental metal exposure to co-select for antibiotic-resistant strains, even in the absence of antimicrobial use. In oral biofilms, such co-selection may enhance microbial persistence, increase resilience to host defense mechanisms, and complicate therapeutic interventions [31,98].

Thus, metal-driven ecological pressure may contribute to a broader shift toward resistant, less responsive microbial communities, reinforcing dysbiotic stability [10].

### 6.3. Disruption of Biofilm Architecture and Quorum Sensing

Heavy metals influence not only microbial composition but also structural and communicative properties of biofilms. Metal ions can bind to components of the extracellular polymeric substance (EPS), modifying matrix rigidity, porosity, and diffusion gradients [50]. Changes in EPS cross-linking may alter oxygen penetration, nutrient transport, and spatial organization of microbial subpopulations [99].

Metals also interfere with quorum sensing systems that regulate biofilm maturation, virulence factor expression, and interspecies communication. By altering signaling molecule production or receptor function, metals may disrupt coordinated community behavior. Such interference can shift biofilm dynamics from cooperative equilibrium toward competitive or pathogenic states [100,101].

Additionally, metals can substitute for essential cofactors in bacterial enzymes, perturbing metabolic pathways. Impaired iron- or zinc-dependent enzymatic reactions may trigger compensatory metabolic adaptations, favoring organisms with alternative pathways or enhanced stress tolerance. Over time, these metabolic shifts contribute to functional dysbiosis even when taxonomic changes appear modest [102,103,104].

### 6.4. Oxidative–Inflammatory Feedback Loops

Metal-driven dysbiosis is amplified through host-mediated mechanisms. Redox-active metals promote reactive oxygen species (ROS) generation within epithelial cells and biofilms. At the subcellular level, redox-active metals—particularly cadmium, lead, arsenic, and mercury—impair mitochondrial electron transport chain function, primarily by inhibiting Complex I and Complex III activity, thereby increasing mitochondrial ROS (mtROS) production [105]. Disruption of mitochondrial membrane potential leads to cytochrome c release and activation of downstream apoptotic and inflammatory cascades [106]. Elevated mtROS activates the NF-κB and MAPK/ERK signaling pathways, amplifying transcription of pro-inflammatory cytokines and further destabilizing redox homeostasis within epithelial and immune cells [106]. Additionally, metal-induced impairment of the Nrf2–ARE antioxidant defense pathway reduces cellular capacity to neutralize oxidative stress, creating a self-reinforcing cycle of mitochondrial dysfunction, ROS accumulation, and inflammatory signaling that sustains the dysbiotic microenvironment [107]. Elevated oxidative stress alters epithelial barrier integrity, increases inflammatory cytokine release, and modifies salivary composition [80,108].

Inflammatory mediators such as IL-1β, IL-6, and TNF-α influence nutrient availability and oxygen tension within the biofilm microenvironment [109,110]. These host-derived changes create secondary selective pressures that further favor inflammation-adapted microbial taxa. Thus, metal exposure initiates a feedback loop: metal stress → microbial shift → host inflammation → altered ecological niche → reinforced dysbiosis [17,111,112].

This bidirectional amplification distinguishes metal-induced dysbiosis from transient ecological disturbances [111]. Over time, the persistent oxidative–inflammatory milieu stabilizes dysbiotic configurations and reduces the likelihood of spontaneous ecological recovery [113].

### 6.5. From Ecological Perturbation to Stable Dysbiosis

The transition from exposure to dysbiosis is gradual and context-dependent. Acute exposure may induce reversible shifts, whereas chronic low-dose metal presence favors long-term adaptation [114]. Repeated micro-exposures—through environmental contact, smoking, dietary intake, or intraoral corrosion—sustain selective pressure across microbial generations [7].

Once resistant consortia become established, ecological inertia may maintain dysbiosis even if exposure decreases [115]. Reduced diversity, enrichment of resistance determinants, metabolic reprogramming, and host-mediated feedback collectively promote a new, less resilient steady state [116].

In this framework, heavy metals act as ecological catalysts that lower the threshold for dysbiosis. They do not function as classical pathogens; rather, they reshape the environmental conditions under which pathogenic or inflammation-associated taxa gain dominance [117].

**Figure 1:**
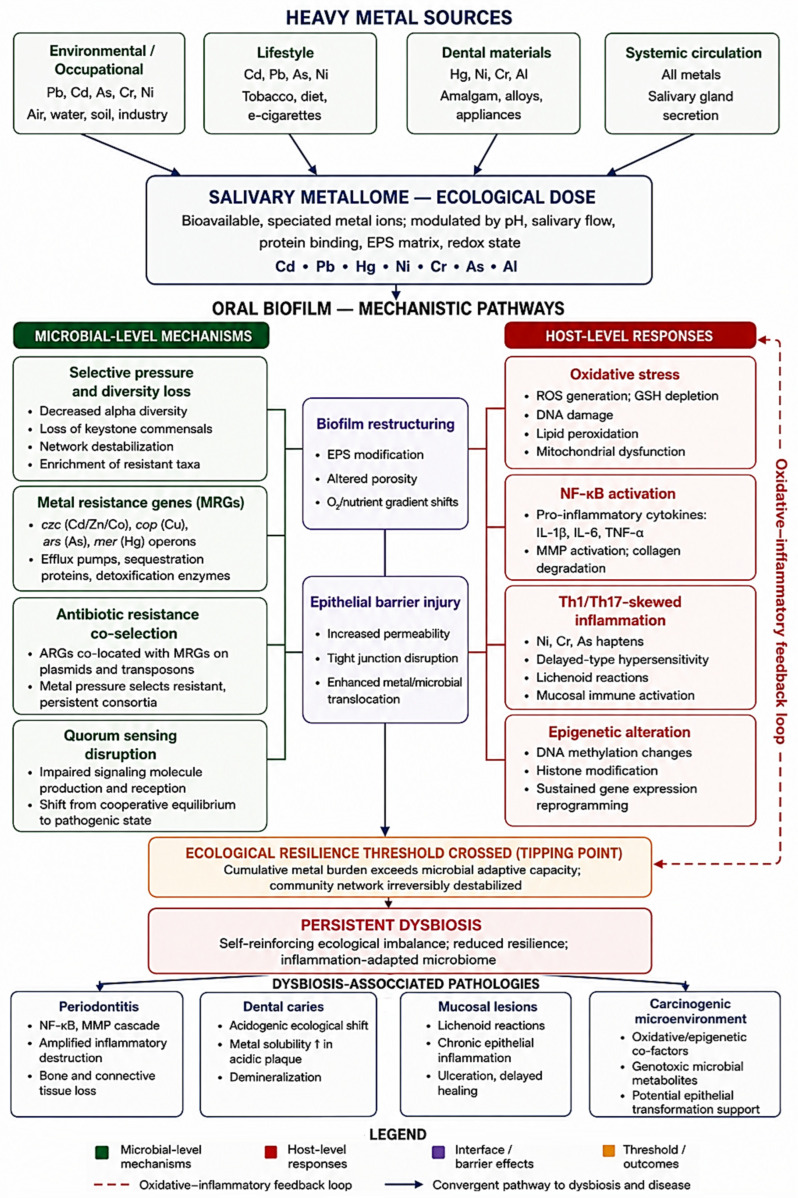
Mechanistic pathways linking heavy metal exposure to oral dysbiosis at the host–microbiome interface. Heavy metals entering the oral ecosystem through environmental, lifestyle-related, dental material–associated, and systemic sources accumulate in saliva as a bioavailable ecological dose. At the microbial level, sustained metal pressure may drive selective loss of diversity, enrichment of metal resistance gene (MRG)-harboring taxa (including *czc*, *ars*, *mer*, and *cop* operons), co-selection of antibiotic resistance genes (ARGs) through shared mobile genetic elements, and disruption of quorum sensing. Concurrently, at the host level, redox-active metals may promote reactive oxygen species (ROS) generation, NF-κB–mediated cytokine release (IL-1β, IL-6, TNF-α), Th1/Th17-skewed immune responses, and epigenetic reprogramming. These interconnected microbial and host-mediated mechanisms converge on biofilm structural remodeling and epithelial barrier disruption. As cumulative metal burden progressively erodes microbial resilience, the system may cross a critical ecological threshold, transitioning toward persistent dysbiosis maintained by self-reinforcing host–microbiome feedback loops. Persistent dysbiosis may contribute to pathological processes associated with periodontitis, dental caries, mucosal lesions, and pro-carcinogenic microenvironments. ARGs, antibiotic resistance genes; EPS, extracellular polymeric substances; MMP, matrix metalloproteinase; MRGs, metal resistance genes; ROS, reactive oxygen species.

The transition from ecological perturbation to persistent dysbiosis can be conceptualized within a resilience–threshold framework in which cumulative metal burden progressively lowers microbial resilience thresholds (the point at which microbial communities lose adaptive capacity) and promotes self-reinforcing host–microbiome feedback loops, as illustrated in Figure 1.

Unlike traditional toxicological models that emphasize dose-dependent cytotoxicity, the resilience–threshold framework proposed here conceptualizes heavy metals as ecological modulators that progressively erode microbial network stability [118]. This systems-level perspective shifts interpretation from direct toxicity toward ecological tipping dynamics and self-reinforcing host–microbiome feedback loops, thereby reframing metal exposure as a driver of sustained ecological destabilization rather than isolated tissue injury [119].

## 7. Dysbiosis-Associated Pathologies: Ecological Imbalance and Potential Disease Implications

Heavy metal exposure is unlikely to function as an isolated causal determinant of oral disease [120]. Rather, current evidence suggests that metals may act as ecological modifiers or amplifiers capable of influencing microbial stability, host inflammatory responses, and biofilm resilience within susceptible biological contexts [121].

### 7.1. Periodontal Inflammation as a Model of Metal-Amplified Dysbiosis

Periodontal disease represents a paradigmatic example of dysbiosis-driven pathology [122]. In health, periodontal biofilms maintain equilibrium through cooperative interactions among commensal and low-abundance pathobionts [123]. Under persistent ecological stress, including heavy metal exposure, this balance may shift toward enrichment of inflammation-associated taxa such as Fusobacterium, Prevotella, and other proteolytic organisms [124].

Metal-driven selective pressure has been associated with reductions in microbial diversity and destabilization of co-occurrence networks, thereby favoring organisms capable of tolerating oxidative stress and inflammatory conditions [125]. Concurrently, redox-active metals may amplify reactive oxygen species (ROS) generation within gingival tissues, potentially enhancing NF-κB–mediated cytokine production and matrix metalloproteinase activation [126].

The resulting microenvironment—characterized by oxidative stress, cytokine release, and collagen degradation—further selects for inflammation-adapted microbial consortia [127]. Thus, periodontal disease may be conceptualized as a potentially self-reinforcing ecological shift in which chronic metal exposure may contribute to lowering resilience thresholds and amplifying inflammatory dysbiosis in predisposed oral environments [124,128].

Importantly, the ecological perspective emphasizes that metals do not replace classical risk factors (such as biofilm accumulation or smoking), but may intensify microbial imbalance in already susceptible niches [7,129].

### 7.2. Dental Caries and Acidogenic Ecological Shifts

Dental caries is driven by functional dysbiosis characterized by enrichment of acidogenic and aciduric bacteria [130]. Heavy metals may influence this process indirectly by altering microbial competitiveness and metabolic pathways within biofilms [131].

Selective pressure favoring stress-tolerant taxa can modify carbohydrate metabolism and acid production dynamics [132]. In parallel, metal-induced oxidative stress may affect enamel integrity and salivary buffering capacity, subtly shifting the ecological conditions that regulate demineralization–remineralization balance [133].

Moreover, acidic microenvironments within cariogenic lesions enhance metal solubility, increasing local ionic availability and potentially reinforcing selective pressure within plaque biofilms [64]. This bidirectional interaction illustrates how dysbiosis and physicochemical changes mutually amplify one another in the presence of persistent environmental stress [134].

Nevertheless, available evidence remains insufficient to support a direct causal relationship between heavy metal exposure and cariogenic progression. Current findings are more consistent with a modulatory ecological role involving biofilm restructuring and altered physicochemical conditions within the oral niche.

### 7.3. Mucosal Pathology and Immune-Mediated Imbalance

Oral mucosal lesions, including lichenoid reactions and hypersensitivity phenomena, reflect immune-mediated responses at the host–microbe–metal interface [135,136,137]. Metals such as nickel and chromium can function as haptens, forming complexes with host proteins that trigger delayed-type hypersensitivity responses [138,139].

Beyond direct immunogenicity, metal-driven dysbiosis may contribute to mucosal pathology by altering microbial metabolites, barrier function, and inflammatory tone [139,140]. Reduced microbial diversity and enrichment of pro-inflammatory taxa may be associated with amplified cytokine signaling, potential disruption of epithelial tight junctions, and maintenance of chronic mucosal irritation [141].

In this setting, dysbiosis and immune activation form a coupled system: community instability intensifies inflammation, while inflammation further modifies the microbial niche [142]. Metals may act as persistent modulators within this cycle, potentially contributing to low-grade immune activation associated with recurrent lesions [143].

These interactions are likely influenced by host susceptibility, immune context, exposure duration, and local ecological conditions, further supporting a multifactorial rather than deterministic interpretation of metal-associated mucosal pathology.

### 7.4. Carcinogenic Microenvironment and Chronic Ecological Stress

Chronic dysbiosis has been increasingly implicated in carcinogenesis through mechanisms involving oxidative stress, epithelial–mesenchymal transition, immune dysregulation, and microbial metabolite production [4]. Heavy metals classified as carcinogenic or potentially carcinogenic compounds may contribute to this process not only through direct genotoxicity but also through sustained ecological perturbation [144,145].

Metal-driven oxidative stress has been associated with DNA damage and epigenetic alterations in epithelial cells. Simultaneously, dysbiotic microbial communities may produce pro-inflammatory mediators and genotoxic metabolites that enhance epithelial proliferation and genomic instability [146,147]. The convergence of persistent oxidative stress, inflammatory signaling, and microbial imbalance may contribute to a microenvironment permissive to malignant transformation [145,148,149].

Within this framework, metals should be viewed as ecological co-factors that amplify carcinogenic signaling cascades through microbiome-mediated mechanisms rather than as isolated mutagenic agents [150].

Nevertheless, current evidence linking heavy metal–associated oral dysbiosis to epithelial transformation remains largely indirect and predominantly associative. The interaction between chronic inflammation, microbial imbalance, environmental exposure, host susceptibility, and carcinogenic signaling is highly complex and context-dependent. Therefore, heavy metals should be interpreted primarily as potential ecological co-modulators within multifactorial carcinogenic processes rather than direct oncogenic drivers.

### 7.5. Beyond the Oral Cavity: Systemic Implications of Local Dysbiosis

The oral cavity communicates continuously with systemic compartments via vascular exchange, immune trafficking, and ingestion of saliva [151]. Persistent oral dysbiosis may therefore contribute to systemic inflammatory burden. Swallowed dysbiotic biofilm fragments and inflammatory mediators can influence gastrointestinal and metabolic pathways, while circulating cytokines derived from chronic periodontal inflammation may affect distant tissues [152,153].

Although causality remains incompletely defined, the concept of an exposome-driven oral–systemic axis underscores the potential relevance of metal-induced dysbiosis beyond localized oral pathology [18]. The oral cavity may serve as an early indicator of broader ecological disturbances triggered by chronic environmental metal exposure [154].

## 8. Biomarkers of Metal-Induced Dysbiosis

If heavy metals function as ecological stressors, dysbiosis represents the transitional state between exposure and clinically manifest pathology. Within this continuum, biomarkers should not be regarded merely as indicators of metal presence, but as measurable expressions of community instability and maladaptive host–microbe interactions [155]. Metal-induced dysbiosis generates a multilayered biological signature encompassing elemental patterns, microbial restructuring, oxidative imbalance, inflammatory activation, and regulatory reprogramming [111]. These domains are interdependent and gain explanatory power when interpreted integratively rather than as isolated parameters.

### 8.1. Salivary Metallomic Patterns as Ecological Pressure Indicators

Total metal concentration provides limited insight into ecological impact [156]. Biological relevance lies not in isolated elemental values but in the configuration of the metallomic profile [157]. Concurrent elevations of cadmium, lead, nickel, or chromium, for example, may reflect cumulative selective pressure capable of disrupting microbial homeostatic equilibrium even when individual concentrations remain below conventional toxicity thresholds [158].

Relative abundance, elemental ratios, and co-occurrence patterns may correlate more strongly with microbiome restructuring than single-metal measurements. In this context, the salivary metallome can be conceptualized as a proxy for ecological pressure intensity [4]. When interpreted alongside microbial diversity indices and host inflammatory markers, metallomic patterns help contextualize whether the oral ecosystem is operating within adaptive resilience or transitioning toward dysbiotic instability [159].

### 8.2. Microbiome-Based Biomarkers of Ecological Imbalance

Microbial signatures provide the most direct readout of dysbiosis. Metal-induced ecological stress may manifest as reduced alpha diversity, altered beta diversity patterns, and reorganization of community structure with loss of keystone commensals [51]. At the same time, taxa capable of tolerating oxidative or metal stress may increase in relative abundance, reshaping interspecies networks and metabolic cooperation [159].

Beyond taxonomic composition, functional alterations may offer deeper mechanistic insight. Enrichment of genes encoding metal efflux systems, detoxification pathways, and oxidative stress responses suggests adaptive reprogramming under persistent chemical pressure [160]. Such metagenomic shifts indicate not merely compositional change but ecological adaptation, which may precede overt inflammatory or clinical manifestations. Accordingly, microbiome-derived biomarkers serve as early indicators of declining network stability at the host–microbe interface [161].

### 8.3. Oxidative Stress and Redox Biomarkers

Redox imbalance constitutes a central mechanistic bridge between heavy metal exposure and microbiome alteration [158]. Salivary markers of oxidative damage—including nucleic acid oxidation products such as 8-hydroxy-2′-deoxyguanosine, lipid peroxidation derivatives such as malondialdehyde, protein carbonylation, and variations in total antioxidant capacity—reflect amplification of oxidative processes within the oral environment [162].

Importantly, these markers signify more than tissue injury. Elevated reactive oxygen species modify oxygen gradients, influence pH dynamics, and alter nutrient availability within biofilms [163]. Such physicochemical changes reshape microbial competitiveness and may favor stress-adapted taxa, thereby reinforcing dysbiosis. Redox biomarkers therefore function as indicators of ecological niche transformation rather than simple measures of cellular damage [164].

### 8.4. Inflammatory and Immune Signatures

Persistent low-grade inflammation frequently accompanies metal-driven dysbiosis. Alterations in salivary cytokine profiles—such as increased interleukin-1β, interleukin-6, tumor necrosis factor-α, and interleukin-8, together with changes in secretory IgA levels—reflect disruption of mucosal immune homeostasis [165].

Inflammation and dysbiosis operate as mutually reinforcing processes. Host-derived inflammatory mediators modify epithelial permeability, oxygen tension, and nutrient composition within saliva, thereby reshaping the microbial niche [166]. In turn, dysbiotic communities amplify inflammatory signaling through pathogen-associated molecular patterns and metabolic by-products [167]. Consequently, inflammatory biomarkers serve as dynamic indicators of an evolving feedback loop in which metal exposure, microbial imbalance, and host response co-amplify ecological instability [17].

### 8.5. Epigenetic and Regulatory Biomarkers

Chronic exposure to heavy metals has been associated with regulatory alterations that extend beyond immediate inflammatory responses [168]. Epigenetic modifications—including DNA methylation changes, histone modification patterns, and microRNA dysregulation—may reflect longer-term biological imprinting of environmental stress [169].

In oral epithelial cells, such modifications can influence barrier integrity, antioxidant defense capacity, and immune responsiveness [170]. Dysregulated microRNA expression linked to oxidative or inflammatory pathways may precede overt tissue damage, representing early molecular evidence of ecological stress [171]. Unlike transient inflammatory markers, epigenetic signatures may capture cumulative exposure effects and sustained host adaptation, offering insight into long-term vulnerability [172,173].

### 8.6. Toward Integrated Multi-Layered Biomarker Models

No single biomarker adequately captures the complexity of metal-induced dysbiosis. A more informative framework integrates metallomic patterns as indicators of ecological pressure, microbiome restructuring as evidence of community response, oxidative markers as reflections of niche transformation, inflammatory mediators as signs of host engagement, and epigenetic signatures as markers of long-term adaptation [17].

Within such a model, biomarkers are not isolated endpoints but interconnected nodes within an exposure–microbiome–host network [174]. Longitudinal investigation is essential to determine whether specific biomarker constellations signify transient perturbation or stable dysbiosis with pathological potential [175]. By embracing a systems-level perspective, salivary biomarkers may move beyond descriptive association toward mechanistic interpretation of environmentally driven microbiome imbalance [176].

The multi-layered biomarker architecture underlying metal-driven dysbiosis is summarized in Table 2.

## 9. Methodological Gaps and Research Priorities

Despite growing recognition of heavy metals as ecological modulators of the oral microbiome, the current body of evidence remains largely associative. Most available studies rely on cross-sectional designs, single-time-point measurements, and isolated metal assessments, limiting the capacity to establish temporality or causality between exposure, dysbiosis, and pathological outcomes. Advancing this field requires methodological refinement, conceptual integration, and systems-level approaches.

Additional limitations include the predominance of small study populations, cross-sectional study designs, heterogeneous exposure assessment methods, and the limited availability of longitudinal investigations capable of capturing temporal microbiome dynamics and resilience trajectories. These constraints may contribute to variability across studies and limit causal interpretation.

A major limitation lies in the heterogeneity of exposure assessment. Salivary metal measurements are often reported as total concentrations without characterization of chemical speciation, binding state, or bioavailable fraction [19,82]. Given that ecological impact depends on speciation and redox activity rather than absolute concentration alone, this distinction is particularly important for comparing salivary metallomic studies, as biologically active fractions may differ substantially despite similar total elemental concentrations. Moreover, real-world exposure typically involves mixed-metal profiles, yet most studies evaluate single elements independently. Incorporating mixture modeling and cumulative exposure indices would better reflect ecological reality.

Microbiome analyses present parallel challenges. Many investigations focus on taxonomic composition without integrating functional metagenomic or transcriptomic data. However, ecological adaptation under metal pressure may manifest primarily through functional reprogramming rather than overt compositional change. Future research should therefore combine taxonomic profiling with pathway-level analyses, including metal resistance determinants, oxidative stress response genes, and metabolic network restructuring. Network-based ecological modeling may further elucidate shifts in community stability and resilience.

Another critical gap concerns temporality. Cross-sectional snapshots cannot distinguish transient perturbations from stable dysbiotic states. Longitudinal designs are essential to determine whether metal exposure precedes microbiome alteration and whether specific biomarker constellations predict progression toward inflammatory or neoplastic pathology [177]. Repeated measures of metallomic patterns, microbial diversity, oxidative markers, and immune mediators within the same individuals would allow construction of dynamic exposure–response trajectories. Furthermore, causal inference remains challenging because most available studies rely on observational associations rather than experimental designs capable of establishing mechanistic relationships. Longitudinal cohort studies combined with controlled experimental systems, including advanced multispecies biofilm models and mechanistic in vivo investigations, will be essential for determining whether heavy metal exposure precedes ecological disruption and contributes to persistent dysbiotic transitions.

Standardization also remains insufficient. Variability in saliva collection protocols, flow rate control, storage conditions, sequencing platforms, and bioinformatic pipelines complicates cross-study comparison. Harmonized methodological frameworks are necessary to reduce technical noise and clarify biological signal. Without such standardization, it remains difficult to determine whether observed discrepancies reflect true ecological variability or methodological artifacts [178].

Experimental models represent another area for development. In vitro systems frequently rely on simplified biofilms or artificial saliva formulations that do not replicate the complexity of the human oral ecosystem. Advanced multispecies biofilm models incorporating physiologically relevant salivary components, controlled redox gradients, and graded metal exposure would provide greater mechanistic resolution. Similarly, integration of in vivo models and well-characterized human cohorts could bridge the gap between molecular mechanisms and clinical relevance.

Finally, integration across biological layers remains limited. Few studies simultaneously assess metallomic profiles, microbial functional signatures, host inflammatory mediators, and epigenetic modifications. Nevertheless, emerging multi-omics investigations have begun integrating oral microbiome composition with inflammatory biomarkers, oxidative stress signatures, and salivary molecular profiles, supporting the feasibility of systems-level approaches [179]. Existing studies suggest that combined biomarker frameworks may improve characterization of host–microbiome interactions compared with isolated biomarkers alone, although comprehensive integration of metallomic, microbial, oxidative, inflammatory, and epigenetic datasets remains relatively limited [180]. Systems biology approaches may help clarify causal pathways. Integration of multi-omics data with ecological modeling could also identify threshold effects and tipping points during the transition from resilience to dysbiosis [181]. Such integrative designs are particularly important for distinguishing adaptive microbial responses from maladaptive ecological collapse.

Beyond mechanistic understanding, these findings may also have practical implications for oral health surveillance and preventive strategies. In populations with elevated environmental or occupational metal exposure, integration of salivary metallomic profiling with microbiome-associated biomarkers may support early identification of ecological disturbances before overt clinical disease develops. Potential interventions could include exposure reduction strategies, targeted salivary screening programs and microbiome-based monitoring of high-risk populations, optimization of oral hygiene measures, dietary approaches aimed at reducing oxidative burden, as well as development of personalized preventive protocols guided by integrated biomarker signatures. Although these applications remain largely investigational, they may contribute to future precision-based approaches for oral health risk assessment and disease prevention.

Future research should prioritize prospective longitudinal cohort studies with repeated exposure measurements and standardized saliva collection protocols. Integration of multi-omics approaches—including salivary metallomics, functional metagenomics, transcriptomics, inflammatory profiling, and epigenetic analyses—may improve characterization of dynamic host–microbiome interactions. In addition, cumulative metal exposure assessment should extend beyond individual elemental measurements and incorporate mixture modeling, ecological dose estimation, and exposome-based analytical frameworks capable of capturing real-world environmental complexity.

In summary, advancing the understanding of metal-associated oral dysbiosis requires movement beyond descriptive association toward mechanistic and longitudinal investigation. Emphasis on speciation-aware exposure assessment, mixture analysis, functional microbiome profiling, harmonized methodologies, and multi-layered biomarker integration will be essential for establishing causal links between environmental metal burden and microbiome-mediated pathology. Only through such rigorous approaches can salivary metallomics and microbiome analysis transition from correlative observation to predictive ecological modeling of disease susceptibility.

## 10. Conflicting Evidence and Limitations

Although heavy metals are increasingly recognized as modulators of oral microbial ecosystems, the available evidence remains heterogeneous and, in some respects, contradictory. Variations in study design, exposure assessment, analytical methodologies, and biological interpretation limit the ability to draw definitive causal conclusions.

Associations between metal exposure and microbiome composition are inconsistently reported. Some studies identify reduced microbial diversity and enrichment of metal-tolerant taxa under chronic exposure, while others observe only modest or context-dependent effects. These discrepancies arise from differences in exposure intensity and duration, population characteristics, and sampling. Furthermore, microbiome responses may be nonlinear, as low-level exposures can induce adaptive changes that do not necessarily result in dysbiosis.

Distinguishing direct metal–microbiome interactions from indirect host-mediated effects remains challenging. Heavy metals can alter epithelial barrier function, salivary composition, and immune signaling, each of which may independently reshape microbial communities. Consequently, observed microbiome alterations may reflect downstream host responses rather than direct microbial toxicity.

Most available studies are limited by relatively small sample sizes, predominantly cross-sectional designs, heterogeneous exposure assessment methods, and insufficient longitudinal follow-up. Consequently, temporal relationships among metal exposure, microbiome alterations, and disease progression remain difficult to establish. Confounding factors such as diet, smoking, oral hygiene, dental materials, medication use, and socioeconomic variables further complicate interpretation and may contribute to inconsistent findings across studies.

Methodological heterogeneity reduces comparability across studies. Variations in saliva collection protocols, analytical platforms, sequencing approaches, and bioinformatic pipelines contribute to variability in both metallomic and microbiome data. Additionally, most studies report only total metal concentrations without accounting for chemical speciation or bioavailable fractions, which limits the interpretation of biological relevance.

Generalizability is further constrained by population-specific factors and small sample sizes, particularly in studies conducted within high-exposure environments.

In summary, although the role of heavy metals in shaping oral microbiome dynamics is biologically plausible, current evidence remains largely associative. Future research should prioritize longitudinal study designs, methodological standardization, and integrative multi-omics approaches to clarify causal pathways and identify thresholds for the transition to persistent dysbiosis.

## 11. Conclusions

Heavy metals should no longer be regarded solely as passive environmental contaminants measurable in saliva, rather, they may also function as ecological modulators of oral microbial community stability. Within a dysbiosis-centered framework, chronic low-dose metal exposure may function as a sustained selective force capable of reshaping microbial structure, functional capacity, and host–microbe signaling dynamics. The resulting community instability may represent an important intermediate ecological state linking environmental burden to inflammatory, degenerative, and potentially neoplastic processes.

Situated at the intersection of external exposure and systemic physiology, the oral cavity operates as a biologically dynamic exposome–microbiome interface. When interpreted alongside microbial, oxidative, inflammatory, and regulatory signatures, salivary metallomic profiles—particularly in relation to metal speciation, bioavailability, and ecological dose—may provide insight into microbial network resilience and adaptive capacity rather than simple exposure status. This systems-level perspective shifts emphasis from isolated concentration thresholds toward integrated exposure–response modeling.

Although current evidence remains predominantly associative, convergent mechanistic and multi-layered biomarker data support the biological plausibility of metal-associated microbiome destabilization. Nevertheless, causal relationships, resilience thresholds, and temporal dynamics remain incompletely defined. Advancing this field will require longitudinal, speciation-aware, and multi-omics approaches capable of distinguishing adaptive microbial restructuring from persistent ecological collapse.

By reframing salivary heavy metals within the architecture of microbiome ecology, this paradigm moves beyond traditional toxicological assessment and supports the potential incorporation of resilience metrics, microbial network stability parameters, and integrative biomarker signatures into environmental risk evaluation frameworks.

## Figures and Tables

**Table 1:** Comparative overview of major heavy metals as ecological drivers of oral dysbiosis: exposure sources, microbiome alterations, host responses, and pathological implications. The table provides a comparative overview of major heavy metals and integrates current evidence regarding their environmental sources, microbiome-level effects, host biological responses, and dysbiosis-associated pathological implications within the proposed ecological framework.

Heavy Metal	Major Exposure Sources	Microbiome-Level Effects	Host-Level Responses	Dysbiosis-Associated Pathological Implications
Cadmium (Cd)	Industrial emissions; tobacco smoke; contaminated food and water	Reduced alpha diversity; enrichment of metal-resistant taxa; upregulation of efflux and stress-response genes	Increased oxidative stress; epithelial barrier disruption; pro-inflammatory cytokine activation	Amplified periodontal inflammation; reduced ecological resilience under chronic exposure
Lead (Pb)	Environmental pollution; contaminated water; occupational exposure	Altered community composition; network destabilization; selective pressure favoring resistant phenotypes	Oxidative damage; immune modulation; altered salivary protein profiles	Increased susceptibility to inflammatory oral conditions; potential contribution to systemic inflammatory burden
Mercury (Hg)	Dental amalgam release; contaminated seafood; occupational exposure	Disruption of quorum sensing; enrichment of mercury-resistance operons (mer); functional metabolic shifts	Reactive oxygen species generation; DNA damage; immune activation	Promotion of chronic mucosal irritation; contribution to carcinogenic microenvironment
Nickel (Ni)	Orthodontic appliances; dental alloys; industrial exposure; tobacco	Selection of nickel-tolerant taxa; biofilm structural modification; co-selection of resistance determinants	Hypersensitivity reactions; Th1/Th17-skewed immune responses	Lichenoid reactions; mucosal inflammation; exacerbation of dysbiotic states
Chromium (Cr)	Industrial processes; occupational inhalation; dental materials	Enrichment of oxidative stress-adapted taxa; metabolic reprogramming under redox pressure	NF-κB pathway activation; epithelial oxidative injury	Sustained inflammatory niche; potential co-factor in epithelial transformation
Arsenic (As)	Contaminated groundwater; dietary sources	Enrichment of *ars* operon–harboring bacteria; altered redox metabolism	Mitochondrial dysfunction; inflammatory cytokine release; epigenetic alterations	Chronic inflammatory imbalance; possible systemic effects via oral–gut axis
Aluminum (Al)	Food additives; environmental exposure; dental materials	Modulation of microbial metabolic pathways; interference with metal homeostasis	Oxidative imbalance; altered mucosal immune responses	Contribution to low-grade ecological destabilization under cumulative exposure

**Table 2:** Multi-layered biomarkers potentially associated with heavy metal-driven oral dysbiosis and ecological monitoring. The table summarizes candidate metallomic, microbial, oxidative, inflammatory, and epigenetic biomarkers potentially relevant for ecological monitoring and dysbiosis-associated risk assessment.

Biological Layer	Representative Biomarkers	Ecological Interpretation	Potential Clinical Relevance
Metallomic Layer	Salivary Cd, Pb, Hg, Ni, Cr, As; metal ratios; mixed-metal profiles	Indicator of ecological pressure intensity; cumulative selective stress on biofilms	Exposure stratification; identification of high-risk ecological contexts
Microbiome Composition	Alpha diversity indices (Shannon, Simpson); beta diversity shifts; loss of keystone taxa	Reduced resilience; network destabilization; dysbiotic restructuring	Early detection of microbiome instability; risk assessment for periodontal or mucosal pathology
Functional Metagenomics	Metal resistance genes (*czc*, *mer*, *ars* operons); efflux systems; oxidative stress response genes	Adaptive microbial reprogramming under metal pressure; co-selection of resistance determinants	Prediction of persistent dysbiosis and reduced therapeutic responsiveness
Oxidative Stress Layer	8-OHdG; malondialdehyde (MDA); protein carbonyls; total antioxidant capacity	Redox imbalance; modification of biofilm niche conditions; ROS-mediated ecological shifts	Indicator of microenvironmental instability; monitoring of inflammatory amplification
Inflammatory/Immune Layer	IL-1β; IL-6; TNF-α; IL-8; secretory IgA alterations	Host–microbiome maladaptation; feedback amplification of dysbiosis	Identification of transition from ecological perturbation to inflammatory pathology
Epigenetic/Regulatory Layer	DNA methylation changes; histone modifications; dysregulated microRNAs	Long-term biological imprinting of chronic exposure; sustained vulnerability state	Potential markers of chronic ecological collapse and disease susceptibility
Integrated Multi-Omics Signature	Combined metallomic + microbial + inflammatory + epigenetic profiles	Systems-level transition beyond resilience threshold; stabilized dysbiosis	Predictive modeling of progression toward periodontal, mucosal, or neoplastic pathology

## Data Availability

No new data were created or analyzed in this study. Data sharing is not applicable to this article.

## Abbreviations

The following abbreviations are used in this manuscript:

Hg	Mercury
Hg^2+^	Divalent mercury
IgA	Immunoglobulin A
IL	Interleukin
IL-1β	Interleukin-1 beta
IL-6	Interleukin-6
IL-8	Interleukin-8
MDA	Malondialdehyde
mer	Mercury resistance operon
MRGs	Metal resistance genes
NF-κB	Nuclear factor kappa B
Ni	Nickel
Pb	Lead
ROS	Reactive oxygen species
Th1	T helper type 1 cells
Th17	T helper type 17 cells
TNF-α	Tumor necrosis factor alpha
8-OHdG	8-Hydroxy-2′-deoxyguanosine
